# Topics of Public Interest

**Published:** 1905-05

**Authors:** 


### TOPICS OF PUBLIC INTEREST.

### Physicians of Erie County Oppose Osteopathy Bill.

A MASS meeting of physicians was held Saturday evening, April 15, 1905, under a call of the Buffalo Academy of Medicine, which read as follows:

BUFFALO ACADEMY OF MEDICINE.

A special meeting of the academy will be held at the academy rooms, Public Library building, on Saturday evening, April 15, 1905, at 8.30 o'clock.

All physicians of Buffalo are invited to be present.

There is at present a bill before the legislature to license the practice of osteopathy in this state. Radical steps are required to prevent the passage of this bill.

It is the duty of every physician to attend this meeting and show where he stands in this matter.

Our representatives at Albany have been invited to attend this meeting.

EDWIN A. BOWERMAN, M. D., Secretary.  ARTHUR W. HURD, M. D., President.

Pursuant to the foregoing about 100 members of the profession, without regard to school or affiliation, assembled at the appointed time and place. In the absence of the president of the academy, Dr. Edward R. McGuire called the meeting to order, stating its purposes, and remarking that the object being outside the province of the academy, a chairman should be named from the profession at large.

Dr. H. U. Williams, of the university faculty, nominated Dr. William C. Phelps, who was unanimously elected chairman. On taking the chair Dr. Phelps stated that the meeting was called to protest formally against the bill to legalise the osteopath. He said that everything that the people of Buffalo could do to stop the passage of this bill should be done.

Dr. Charles G. Stockton, having been called upon by the chair, explained that the bill under discussion originally introduced in the state senate by Senator Davis, after having been amended, or pretendedly so, the result of hearings and conferences, had been reported out of committee and was now on the senate calendar with the probability of being voted on early next week.

Dr. Stockton then requested Dr. Hopkins, who was more familiar with the bill since it had been so complicated by so-called amendments, to read its essential sections. Dr. Hopkins complied with the request and also read from sections of the public health law, showing inconsistency between the two measures.

Dr. Stockton, resuming, said the chief aim of the osteopaths is to become legalised practitioners of medicine. That is the main object of the bill. I think the bill is absurd and should be defeated by all means. The impression prevails in Albany that the osteopaths have consented to amendments which remove our objections to the bill, but this is an entirely mistaken notion. The bill has gone to a third reading and is very likely to pass and go to the Governor, with the impression that the physicians of the state do not object to it in its present form. But I feel that we should object to it in its present form far more strenuously than we did to the original bill. The bill provides no age requirement, no moral character test, no preliminary education, no professional acquirements of any kind for those now in practice.

Dr. Stockton branded the whole measure as absurd and said further that the amendments only made it the more so. "The impression seems to prevail among the members of the assembly," said Dr. Stockton, "that the physicians, or the majority of them at least, do not object to the measure, but such is not the case. Only by united efforts can we defeat the bill." In closing, he read a memorial to the legislature protesting against the passage of the bill in question and moved its adoption.

Dr. Charles Cary seconded the motion, and said: We all disapprove of this bill. I regret very much that the members of the legislature, invited as guests to be present at this meeting, have not appeared. Those who have watched the growing restricttions which have been placed on physicians would extremely regret to see this door thrown open in this way. It would be a crime in my opinion and an absolutely retrogressive action on the part of the state. I think the bill is most vicious and should receive our most hearty condemnation. I cannot express myself too strongly on the subject.

Dr. Walter D. Greene, health commissioner, said: We are all of the same mind. I am very sorry that some of our legislative representatives are not here. It certainly could not help but have an influence upon them, because of the large number of physicians who are here tonight, representative men of the medical profession without regard to school. I wish to add my voice and influence toward the defeat of this bill. I think some of our representatives in this county are pledged to it, and it would be impossible to get them to vote against it. If it passes both houses, I think that our efforts should be entirely centered upon the Governor, who I believe could be made to see quite clearly the fallacy of the law which would permit the practice of medicine without proper training.

The chairman said: There is a petition being circulated about the room, containing Dr. Stockton's preamble and resolution, which it is desirable that every one present should sign.

Dr. Frederick H. Millener said: I am glad that there are so many here tonight to oppose this bill, for it seems to me that there are a good many of the newspapers, including some of the medical journals, that are in favor of the bill. They are seeking to make a business of the practice of medicine without knowing or caring to know what is in it, which reminds me of the bible I saw the other day in the court house carefully sewed up in white cloth, and used to swear witnesses upon, who probably knew nothing and cared less what was in it. It was simply a business matter. I am against this bill, and in favor of these resolutions.

Dr. F. Park Lewis said: It is hardly conceivable that a bill so vicious in its intent could ever receive the endorsement that it seems to have received on the part of the legislators of this state. It is a matter of very grave importance, not alone to the profession, but to the entire citizenship of the state. It means that a large number of thoroughly unqualified men are to be admitted to all the privileges of qualified physicians, and that the struggle we have all passed through in raising the standard of medicine is to go for naught. It does not seem possible that it can pass and yet from intimations that have come to us it looks as though it is a very grave danger with which we are threatened. I take great pleasure in endorsing the resolutions, and I trust that a united effort may be made to prevent the passage of this thoroughly vicious bill.

Dr. Ernest Wende said: I do not care to express my feelings; but if a man wants to practise osteopathy, vitapathy, Christian science or some other fake, I think he could do it with a good conscience if he was legalised. He would not rub where drugs were indicated, he would not pray to remove organic diseases. I certainly oppose the bill. Upon the plea that blacksmiths and barbers have been licensed, the osteopaths also want to be licensed. If the osteopaths are licensed, why every trade will have a right to be licensed, and we will have no practitioners qualified to practise medicine. I hope that argument and common sense will prevail in the legislature.

Dr. J. W. Grosvenor said: Of the osteopaths who are practising at the present time, they are either legal practitioners or they are not. If they are not, they are asking to be legalised without meeting the requirements of the medical law governing the practice of medicine. They should be required to pass the same standards that we have all had to pass. They are asking for class legislation pure and simple. I believe we have good grounds for strenuously opposing the bill. Personally, I am most heartily in sympathy with the protest against the passage of this bill.

Dr. Henry R. Hopkins said: I have appeared in Albany already in opposition to this bill, when some member of the committee asked if it was not a fact that we were opposing the bill because we were jealous of these osteopaths and feared that they would get our business away from us. That represents the high level—are there any reporters here? (A voice, 'Yes'). Good, that gives me further confidence. The first thing that we must do is to get the fact impressed upon the people that, viewed from a purely mercenary standpoint, of all men on earth the medical profession are the only people who can welcome the osteopath and the fakes of all kinds, because they always bring grists to our mill, the maimed to be healed and the sick to be cured. But, happily, we are actuated by loftier purposes.

There is one other fact that is exceedingly important that we should get into the public conscience, because we are here pleading for the upholding of the public health and the cause of preventing sickness. And the public throughout the state thinks it is simply an academic question which amounts to little or nothing. That may seem an extravagant statement, but it is true. The legislators are alert when the question of a few dollars is involved, as witness the wrangle over the amendment to the bill regulating savings banks investments, but when it comes to licensing two or three thousand persons to take the whole responsibility of treating acute diseases they, or some of them, seem indifferent to the training necessary to meet such weighty responsibility. Last year we had in this state 127,000 unnecessary deaths. Does it not require the highest training to cope with such an enormous problem as this fact presents?

This is a great crisis in the history of the state from which it is possible for the medical profession to save it, save it from the intolerable disgrace of having put on its statute books a law which says that the people of the state, in the intelligence of the twentieth century, declare to the world there is no such thing as medicine or surgery; and that is what this bill declares. We will license people to take care of the sick, but say to them, do not practise medicine or surgery. In all seriousness and solemnity there never before was a time when it was possible to save the great state of New York from more terrible degradation,—a disgrace which will make it the laughing stock of all the intelligent people of the world.

Dr. Conrad Diehl, former mayor, remarked: I have listened with a great deal of interest to what has been said. I think we should do all that lays in our power to kill this bill. We owe it to our clientage. I feel that it is our solemn duty to the public. It would be a disgrace to allow these men who have no education whatever to go out among the sick and practise at their will. Dr. Diehl said the osteopaths were great advertisers and that through their advertisements, something not resorted to by legitimate physicians, they would gain many patients.

Dr. James M. O'Neill made a brief address against the measure and then read a prescription that had been given by an osteopath only a day or two before to a woman patient. The prescription called for several different drugs. That, Dr. O'Neill said, was contrary to the teachings even of osteopathy and if they were doing this already what would be the results after the passage of the Davis bill? He also cited a case of tubercular disease of the elbow joint that had been manipulated by an osteopath, causing extensive suppuration above and below the joint, which Dr. O'Neill had treated afterward by surgical intervention.

Dr. Matthew D. Mann said: I wish to call attention to the extreme unfairness of this bill to the medical colleges. The state now enforces the law that every man in order to practise medicine, shall have studied four years, taken a prescribed course of lectures, and passed examinations in certain subjects. Medical colleges have invested millions of dollars, and practitioners have invested very large sums of money and spent their time as well in complying with these advanced standards. And now the state proposes to pass a law admitting a lot of men who have not complied with any of these laws, who have not studied for four years, nor done anything that the law says the rest of us must do, allowing them to practise medicine and making them equal to those who have met these very proper conditions. It is radically wrong and entirely unfair to the medical profession. If a man wishes to practise osteopathy let him take his four years' course and otherwise comply with the present medical practice law, and then he may practise osteopathy if he desires.

Dr. Bernard Bartow said: The public does not demand this measure. It is simply a demand for legislation by a class that expects to receive the benefits. And it is a mistake from every point of view.

Dr. A. L. Benedict: If the osteopaths want to organise and be legislated about, it seems to me that they should have that privilege, the same as the barber, the manager of a Turkish bath or a plumber or anybody else; but we should limit our attention pretty closely to that section of the bill in which the limitations of the osteopath are set forth, and see that their powers are safely and properly limited. There should be sufficient exclusions in the bill to protect the people. For example, they should not have the power to sign certificates of birth, death or contagious diseases.

Dr. Lucien Howe said that he thought it was puerile to pose as benefactors of the human race in opposing the bill. It looks queer, and is likely to queer the influence of the medical profession with the legislators. He felt that it would be an unfortunate bill if passed, and that its effect would be to greatly lower the general tone of the medical profession. He urged that direct personal efforts should be made with the Erie county legislators, especially Senator Davis, the father of the bill, to convince them of the injustice and error of the bill.

Dr. Stephen Y. Howell made a forceful address in opposition to the bill in all its essentials, urging immediate interviews with the Erie county representatives as well as the employment of all reasonable means to inform the members of the legislature throughout the state of the attitude of the profession toward this bill. The course pursued should be firm, dignified, and prompt.

Dr. William C. Krauss indicated the importance of personal and other appeals, in addition to our own senators and assemblymen, to Senators Stevens, l'Hommedieu, and Fancher; to Speaker Nixon, and in particular to the Hon. James C. Sheldon, chairman of the assembly committee on public health. He felt sure that these gentlemen would listen attentively and respectfully to any such appeals, and that such action assuredly would prove of great benefit.

Dr. William Warren Potter said: All the professions except divinity are under the supervision of the board of regents. To ask that board to administer a law that deals with osteopathy, which is only a kind of mechanical treatment, similar to massage, seems to me to be giving that learned body of men a low order of work. It reduces the dignity of the state education department and places it at a disadvantage. It was never intended that it should supervise the licensing of barbers, plumbers or massagists,—all respectable callings, but not requiring higher education. The standard of medical education now reached in this state, and to obtain which the profession struggled so long against a high tide of opposition, should not be lowered, nor should it be endangered even by attaching such duties as this bill contemplates, to the regents of the university.

To become a physician and practise medicine in the state of New York it is required that the candidate be 21 years of age, of good moral character and a student at a medical college for at least four years. Now, if the osteopaths wish to come through that door, there can be no possible objection. A physician properly educated in the essential branches of medicine will not be likely to go wrong in therapeutics.

Remarks of like tenor were make by some others, and then Dr. Mann moved to refer the memorial and protest to a committee to embody in it any suggestions of importance which the discussion had developed, and to send it to the legislature with the signatures of those present attached, and any others that could be secured in season to become effective. The chair appointed Drs. Charles G. Stockton, F. Park Lewis, and Henry R. Hopkins to complete the memorial and protest in accordance with Dr. Mann's motion.

---

The memorial and protest as amended and sent out reads as follows:

TO THE LEGISLATURE OF THE STATE OF NEW YORK.

*Senators and Members of Assembly:*

We, the undersigned physicians of Buffalo and Erie county, present at a meeting held Saturday, April 15, 1905, at 8.30 p. m., at the chambers of the Buffalo Academy of Medicine, having carefully considered the amended senate bill (third reading No. 441), which has for its chief purpose the licensing of several thousand so-called osteopathists now practising medicine in the state of New York,—giving them legal power to practise in all branches of medicine, with restrictions only as to methods of treatment and admitting these men and women of little training, that little being for the most part erroneous and dangerous in character, hence worse than no training, to the privileges of physicians,—desire to enter our protest against the passage of this bill.

We observe that the bill in question permits all so-called osteopaths to sign birth and death certificates; to practise obstetrics and gynecology; to care for eye, ear, nose and throat troubles, as well as heart and lung diseases, including the great army of the tubercular,—which physicians are only just succeeding, through a campaign of education and the liberality of the legislature, in helping to a proper prevention and cure; to direct and manage the insane and mentally defective; to take part in the control of contagious and infectious diseases; to care for the many sick children, who even now from unscientific feeding, go to swell terribly our mortality rate; and to do harm through their ignorant blundering in numberless other ways.

We regard the University of the State of New York,—the education department,—as being the administrative head of the professional education interests of the Empire State, and believe it should not be humiliated by having added to its otherwise dignified duties, the responsibility of controlling interests, conducted on commercial lines, that are foreign to its purposes and beneath its high office.

The osteopath is already a physician or he is not a physician; if he is a physician he needs no class legislation such as is proposed by this bill; if he is not a physician the door already is open to him to become one through the public health law; otherwise, he should not be permitted to assume the duties and privileges that belong to the profession of medicine.

We regard the bill not only as being a menace to the public health, but as a serious embarrassment to our aims in raising the standard of professional qualifications as well as a measure so bad in its effects and even so criminal in its possibilities, that we feel impelled to denounce it and we call upon the elected representatives of the people of the state of New York in senate and assembly to labor for its sure defeat. As amended it is even more objectionable than in its original form.

The following resolution, embracing the essentials of this protest, was passed at the meeting by unanimous vote:

*Resolved,* That the physicians of Buffalo and county of Erie, whose names are hereunto attached, regardless of school or affiliation, denounce Senate bill (third reading No. 441, etc.) known as "An act regulating the practice of osteopathy in the state of New York," as a pernicious measure. We believe that its passage would lead to evils so great as to be criminal in character; we recognise that its main object is to legalise the practice of the thousands of osteopaths now practising in this state, rather than to provide for the education of others in the future, and we respectfully, but most positively and forcefully request the representatives of the people of the entire state in senate and assembly to exhaust all reasonable means to defeat the aforesaid bill.

We present this petition and urge this protest without prejudice against the osteopath personally and altogether aside from the merits or demerits, if there be any such, of the system employed.

(Signed)

Chas. G. Stockton,
C. C. Frederick,
Bernard Bartow,
W. C. Phelps,
Edwin A. Bowerman,
E. R. McGuire,
Charles Cary,
J. M. O'Neill,
Matthew D. Mann,
Henry N. Miller,

Gordon Potter,
Regina Flood-Keyes,
A. R. Satterlee,
Frederick H. Millener,
Walter D. Greene,
A. W. Bayliss,
Irving P. Lyon,
L. O. Thoma,
Henry P. Frost,
Chas. P. Eller,

E. S. Wilson,
Arthur G. Bennett,
George J. Haller,
J. B. Croff,
Louis J. Beyer,
Irving M. Snow,
G. R. Trowbridge,
Francis W. McGuire,
F. Park Lewis,
J. S. Smith,
Eugene A. Smith,
Theodore M. Leonard,
H. R. Trick,
Francis M. O'Gorman,
Eli H. Long,
Herbert U. Williams,
J. W. Grosvenor,
DeWitt H. Sherman,
William Warren Potter,
Stephen Y. Howell,
Wm. C. Krauss,
L. J. McAdam,
A. J. Colton,
H. R. Hopkins,
Ernest Wende,
E. H. Tweedy,
L. E. Curtice,
Charles S. Jewett,
G. A. Himmelsbach,
Renwick R. Ross,
Lee Masten Francis,
William T. Getman,
Burton T. Simpson,
Edward C. Mann,
Conrad Diehl,
Wm. G. Ring,
E. J. Cook,
Maud J. Frye,
Wm. Ward Plummer,
Nelson G. Russell,
Thomas J. Walsh,
George W. Seitz,
Charles E. Abbott,
Willis G. Gregory,
Charles Weil,
W. F. Beck,
T. H. McKee,
H. C. Rooth,
Julius Ullman,
Albert E. Woehnert,
F. B. Rasbach,
A. L. Benedict,
Thomas F. Dwyer,
W. Harry Glenny,
H. R. Gaylord,
James W. Putnam,
Gustav A. Pohl,
W. H. Macey,
John B. Frisbee,
James Stoddart,
M. Hartwig,
Jacob S. Otto,
Charles A. Clements,
Franklin W. Barrows,
J. C. Wheeler,
Lucien Howe,
E. D. Putnam,
R. Langford Patteson,
Alex. Allan,
F. A. Burghardt,
R. Kimpton,
G. H. Westinghouse.

The following names of physicians unable to be present were secured to this petition and protest after the adjournment of the meeting:

B. J. Maycock,
J. T. Cook,
D. B. Stumpf,
Geo. R. Stearns,
E. P. Hussey,
Geo. R. Critchlow,
Fred D. Lewis,
Geo. T. Moseley,
DeLancey Rochester,
N. L. Burnham,
Roswell Park,
E. L. Bissell,
Henry W. Lattin,
Jane W. Carroll,
Electa B. Whipple,
Mary Huntley,
Mary I. Denton,
Jane N. Frear,
Lillian Craig Randall,
Cora B. Lattin,
Marian Marsh,
Frederick Parmenter,
Herbert N. Squier,
Victor M. Rice,
D. W. Harrington,
Henry R. Lohnes,
Robert F. Sheehan, Jr.
Lesser Kauffman,
E. J. Gilray,
Douglass H. Smith,
J. R. Ferguson,
John L. Van de Mark,
E. M. Anderson,
H. M. Seltes,
Leonard Reu,
Robert Mehnert,
Katherine Munhall,
Frederick S. Brickell,
John Parmenter,
Claude S. Johnson,
Marshall Clinton,
Thomas B. Carpenter,

Myrtle A. Hoag,
Eva Mead,
Ward Saltsman,
F. J. Carr,
J. H. Carr,
C. J. Carr,
A. F. Zittel,
Nelson W. Wilson,
Allen A. Jones,
A. A. Hubbell,
Chauncey W. Grove,
John F. Fairbairn,
Glenn L. Whiting,
John G. Morris,
John C. Plain,
John H. Burke,
Herman W. Schlappi,
Elizabeth (Schugens,
Lorenzo Burrows, Jr.

---

### Dr. Roosa Asks that the Title of Doctor of Medicine be Safeguarded.

At the annual dinner of the Society of Medical Jurisprudence, March 11, 1905, at the Hotel Astor, New York, Dr. D. B. Saint John Roosa made a plea for the safeguarding of the medical profession against an invasion of people who were trying to obtain a legal right to the title of "doctor," without the years of study and labor by which a regular physician obtained his degree. There was room for coöperation between the physicians and lawyers in preventing this, he said. "Opticians, massage persons and osteopathists," he declared, "had no claim to the physician's distinctive title."

The country was all wrong on its educational system, he continued, particularly as to physicians. There seemed to be a conspiracy among the heads of educational institutions to drive physicians from all part in college life, he said, because of the length of time necessary to pass through a college training, as the intended physician could not afford to take four years for college and many other years for medical training and hospital work. The English academic system should be abolished, he suggested, and the German training substituted. He further said:

A physician must have a degree conferred on him by a recognised institution, and then must pass the examinations prescribed by the state board before he is allowed to practise. If these people are allowed to assume the title of "doctor," without having gone through the preliminary years of study and labor which we have had, what will become of the profession? A man goes to Albany, and because he knows about the fitting of glasses thinks he ought to be called doctor. Yet there never was a discovery made about the treatment of the eyes which was not made by an oculist. There are our friends, the massage artists and the osteopathists. Why—you might as well admit the seventh son, who has mystical healing power, or the man who cures heart disease by dislocating a vertebra in ten seconds. You lawyers owe something to our profession,—stand with us in this; see that what we have acquired by hard work and study,—the right to practise medicine,—is not taken away from us.

---

### Paper Milk Bottles.

A SANITARY REFORM WHICH PROMISES MUCH.

CONSUMERS of milk who have come to appreciate the value of purity and freedom from infection, will be interested in an idea that originated in Philadelphia. Every one who has studied the matter carefully knows that there are several ways in which milk may become contaminated. If the dairy farm is an ideal one, if the fluid is promptly cooled, if its temperature remains low during the period of transportation, if the city dealer into whose hands it passes on arrival is both honest and intelligent, there still remains a source of possible mischief. Some of the milk which is bottled before distribution may be injured by a lack of thoroughness in cleaning the glass receptacles after previous use. It is against that particular piece of carelessness that it is now proposed to guard by discarding the present style of bottle altogether and replacing it with another, which, like the cheap wooden plates sometimes provided for picnics, shall be used only once. The new bottle is to be made of heavy paper or pasteboard, manufactured out of spruce pulp. Dr. A. H. Stewart, bacteriologist of the board of health in Philadelphia, conducted a series of tests with it, and reports approvingly upon its qualities.

Several previous attempts to make a paper bottle have failed from various causes. One trouble resulted from moulding the article out of pulp, and another from the thinness of the finished product. Again, the flavor of the wood would sometimes be imparted to the contents, and the cost of manufacture was high enough to be objectionable. In the latest experiment most of these difficulties seem to have been overcome. The bottles are stamped out of heavy three-ply paper, and a conical shape is given to them to facilitate packing for shipment in nests. The bottoms have a double thickness, and their edges are locked in such a way that pressure from above adds to their strength. It is said that a weight of two hundred pounds may be put on a bottle without crushing it. The cover is stout, and has protruding lips for convenience in removal. Glue is used in fastening the overlapping edges of the body, but a coating of paraffine prevents it from affecting the taste of the milk and renders the bottle waterproof. Sterilisation by exposure to a temperature of 212° F. is the final operation to which the receptacle is subjected. It is intended to have half pint, pint and quart sizes.

Dr. Stewart sent to each of several Philadelphia milk shops a bottle of the new kind and one of the old sort, with a request to fill both and send to him. On receiving them he put them on ice. Very often the glass bottle would show signs of leakage, but the paper bottles used remained perfectly tight. In every instance fewer organisms were discovered in the paper bottles than in the glass ones, the ratio being about 1 to 4. He found that "certified" milk kept two days longer in the paper bottles than in the glass ones. How faithfully these results would be parallel if the new device came into general use commercially is not easily predicted, and it is impossible to say what unforeseen defects it may yet develop. Dr. Stewart is highly enthusiastic, however, and on the strength of his verdict a company has been organised to manufacture the paper bottles. Within a few months, Dr. Stewart says, it will have ten machines in operation, each producing 20,000 bottles a day.

Advocates of the new scheme insist that it possesses many minor advantages. A paper bottle weighs two ounces, whereas the glass one holding a quart weighs twenty-four or more.

The carrying capacity of a delivery wagon would be greatly increased,—almost doubled, they say,—because the driver would have no old bottles to collect. The dealer would be subjected to no loss through breakage or the stealing of empty bottles. The wholesale cost of glass bottles is about three cents for pints and five cents for quarts. It is estimated that the paper bottles will cost not more than $8 or $10 a thousand, or not to exceed a cent apiece. In view of the compensations which are expected to attend their use, Dr. Stewart thinks that milk dealers would not be warranted in raising their prices in consequence of substituting the new bottle for the old. However, one important effect which he anticipates from the innovation is that the operation of bottling will be transferred from the headquarters of the city dealer to the dairy farm. Heretofore the danger of breaking during shipment has been a formidable obstacle to such a change, which is extremely desirable from sanitary considerations, and possibly that obstacle may now be removed.

The operation of washing returned milk bottles is today conducted with various degrees of thoroughness. In instances, no doubt, it is well done. Nevertheless, many shocking stories, which probably have a good foundation, are told about the carelessness of lazy drivers of city milk wagons. It is said that they often refill dirty bottles without cleaning them at all. Even when the bottles are brought back to the milk shop to be refilled, the task of preparing them for fresh service is often performed so negligently that they might as well have been let alone entirely. Obviously, if a milk bottle is discarded forever after doing duty once, there cannot be any risk to health from this source.—*The Tribune.*

---

### Municipal Dentists.

MUNICIPAL dentists are appointed and paid for by many of the large towns and cities of Germany. In Strasburg, for example, 2,666 children were examined last year, 699 teeth were filled and 2,912 were extracted. The method of work is simple. The teacher brings his class to the dentist, who examines each mouth quickly and marks on the card each child has brought whether treatment is necessary. If so, the child must come again on a Saturday. Russia is also joining in this movement, and has already fitted up nine such institutions in Saint Petersburg alone. And why not, or rather why so late in coming, one might ask. If it is true that, generally speaking, good teeth are necessary to good health and long life, and if, also, a large and growing proportion of citizens has not good teeth, then it follows that the fact is one of public concern. Is it not, for instance, of as much importance to the community that workmen should have good masticating and digesting powers as that there should be $20,000,000 city halls, public parks, expositions, etc. This little, or large, realisation of preventive medicine has so far got into our American minds that we have ordered the soldiers' teeth to be attended to and his governmental service by so much enhanced. But the soldier is at last paid by the civil worker, and as to his teeth and service we are entirely indifferent.—*American Medicine.*

